# Theosophy and Mrs. Besant

**Published:** 1891-10-24

**Authors:** 


### The Doctor's Armchair.

### THEOSOPHY AND MRS. BESANT.

A man would be a prodigy who should object to the extension of human knowledge. There can be no reason whatever why anybody should object. Similarly a man would be mad who should refuse to believe in a miracle if he himself saw a dead body that had been buried for a considerable length of time actually and certainly brought to life again. Professor Huxley warns the public against too readily deciding that certain things affirmed are untrue because they are, or appear to be impossible. The most scientific man in the world, and the most sceptical, cannot help believing when he is confronted with evidence of a really convincing kind. Some men believe on slender evidence; others on strong: but every man, be his name Huxley, or Hume, or Voltaire, must believe when the evidence which is presented to him personally is actual and complete.

Mrs. Besant has travelled far from the scale of doubt, and now finds herself in the scale of faith. Formerly she could not believe what the whole Christian world believed: now she believes so much that the most credulous Christian finds himself a sceptic compared with her.

What is Theosophy? It is not easy to frame a definition which will satisfy everybody, but the following recipe will give a mixture which will be near enough in composition for all practical purposes:

Take of

| | |
|---|---|
| Conveyed Christianity ... ... ... ... | 2 parts. |
| Asiatic Mysticism ... ... ... ... ... | 2 " |
| Crude Metaphysics ... ... ... ... | $1\frac{1}{2}$ " |
| Pure Twaddle ... ... ... ... | $94\frac{1}{2}$ " |
| | 100 |

Mix thoroughly in a brass mortar with a brass pestle, and evaporate. The resulting vapour will be Besanto-Olcottian Theosophy.

A good many people are declaring themselves adherents of Theosophy; and a good many more are curious about it. So far as appears, there is nothing in it which threatens immediate moral harm: but there is something in it which seems to lead to impairment of the intellect: and impairment of the intellect which affects a large number of persons, especially if those persons belong to the intellectual classes, is both a public and a private misfortune.

There is no intention here of debating the question of Theosophy on general grounds. Few things would be more unprofitable than such a discussion. People will take sides in Theosophy exactly as they will in considering the question of the origin of evil; that is to say, every man will be entirely guided by his own peculiar temperament, and not at all by the actual logic of the situation. But it happens that Mrs. Besant has herself furnished us with a practical test whereby to put to the proof her own character as a discriminating and judicial intellectual force. Mrs. Besant it is plain is a clever woman, as a woman. But the question is, Has she a powerful and judicial intellect, capable of weighing facts and evidence, and deciding on the facts and evidence without being influenced by her own subjectivity? Or, is she merely, after all, a clever and imaginative woman, who yields herself up to the influence of words, and to the deliquium, as Carlyle calls it, of vague, insubstantial, and often unscientific and illogical imaginings and dreams? A medical test, which she herself furnishes in a letter to the *Times*, compels the medical mind to hold a poor opinion of Mrs. Besant's scientific intellectual capacity.

Mrs. Besant goes back to Paracelsus, and his cures, which she says were the marvel of Europe; and she quotes Erasmus as a witness in favour of wonders wrought in incurable cases. Now Paracelsus was a clever man in his own way, and his common sense undoubtedly enabled him to practise with some success as a physician in a medically hide-bound age. But the idea that he added anything at all of an important kind to the permanent resources of the medical art, is a pure delusion. As for Erasmus—as a witness in favour of cures wrought on the incurable—one might as well quote Mrs. Besant herself as a scientific expert in practical physiology. The fact is, that Erasmus knew nothing whatever about either disease or its cure. He was above all things a man of letters; and no doubt was justified to a certain extent in the contempt he entertained for the orthodox medicine of his time. But the notion that he had scientific capacity for deciding the question of the value of magnetism as a method of medical treatment is simply absurd.

Mesmer, according to Mrs. Besant, was a "cruelly used genius," whose contentions are now being verified and carried out by Dr. Charcot and his colleagues. One would like to know on what evidence Mrs. Besant pronounces Mesmer a genius; and also why she considers that he was cruelly used. Mesmer professed to have discovered a force which he called animal magnetism, and by which he cured diseases. Now, either there is such a force as animal magnetism, or there is not: either Mesmer had a definite knowledge of such a force or he had not. But this much, at any rate, is certain about Mesmer, that at first he was taken at his own word. All Paris welcomed him and his work, the members of the medical profession giving him their support, as well as the general public. But, of course, if there were such a definite thing as animal magnetism, and if it could be definitely understood, controlled, and utilised for the cure of disease by one man, Mesmer, then it could also be understood, controlled, and utilised for the cure of disease by other medical men. Not unnaturally, therefore, the physicians and the public of Paris asked Mesmer to divulge his secret; nay, they went so far as to procure for him the offer of an annual pension of 20,000 livres for it—a large fortune in those days. But Mesmer had no secret to divulge. Naturally and properly, therefore, he fell into disrepute, and ended his days in merited obscurity.

Mrs. Besant informs the readers of the *Times* that Charcot and his colleagues are at the present moment carrying out and verifying the contentions of Mesmer at the Salpetriere. Who says so? Certainly not M. Charcot himself. Charcot admits that a certain measure of control can be obtained by persons possessed of one kind of nervous endowment over persons having another kind of nervous endowment; and this power, with its effects, he calls hypnotism, because it can produce hypnotic effects upon patients—that is, it can put them to sleep, it can diminish or take away their consciousness. On the other hand, it can exalt their sensibility, and make them obedient to the stronger nervous influence of their hypnotising director. But M. Charcot does not call this animal magnetism, and if he did the name would be of little consequence. What is of consequence is that M. Charcot does not affirm that this power differs in kind from that which any strong personality exercises over a weaker personality. It does not differ in kind from the power and authority which the firm parent exercises over the wayward child, the enthusiastic preacher over his congregation, or the intelligent doctor or warder in a lunatic asylum over his hapless patients.

Moreover, M. Charcot does not profess to work miraculous cures with his hypnotism. Other medical men in France may and do, but Charcot does not. He uses hypnotism merely as a means of bringing under rational control highly sensitive, wilful, and inconsequent nervous temperaments. This rational control, exercised for a sufficient length of time, is advantageous to such temperaments, and often results in marked improvement of health. But far greater results than these are produced every day by the doctors and attendants of lunatic asylums in the improvement and cure of actual lunatics. The asylum doctor does not resort to hypnotism; he uses firm, persistent, and absolute control. The will of the lunatic learns to yield; it practises yielding day by day, and hour by hour. With the practice of obedience the power of self-control is restored; and with self-control sanity gradually returns. Hypnotism is nothing more than a variety of the large and powerful control which one order of nervous temperament exercises over another.

The medical test shows that Mrs. Besant has still a very great deal to learn. She has lived in books and consorted with enthusiasts too exclusively. If she had lived for a few years in hospitals and lunatic asylums; if she had calmly and judicially observed the effects of various diseases and physiological abnormalities upon the bodies and minds of all sorts of men and women; if she had noted how the calm and judicial intellect always rises to the summit above every form of faith, imagination, enthusiasm, disease, and vagary of the human mind force, and steadily holds the balance among them by undoubted divine right, she would not have been so ready as she is to-day to believe the incredible, and to go to martyrdom for the impossible, simply because it is impossible. Science—that is, knowledge and reason—would still rule the world, even though Mahatmas and faith-healers should perform as many great and undoubted miracles as they now believe and proclaim great and undoubted delusions.

